# A Valuable Biochar from Poplar Catkins with High Adsorption Capacity for Both Organic Pollutants and Inorganic Heavy Metal Ions

**DOI:** 10.1038/s41598-017-09446-0

**Published:** 2017-08-30

**Authors:** Xia Liu, Ju Sun, Shengxia Duan, Yanan Wang, Tasawar Hayat, Ahmed Alsaedi, Chengming Wang, Jiaxing Li

**Affiliations:** 1 Institute of Plasma Physics, Chinese Academy of Sciences, P.O. Box 1126, Hefei, 230031 P.R. China; 2 University of Science and Technology of China, Hefei, 230026 P.R. China; 3 Collaborative Innovation Center of Radiation Medicine of Jiangsu Higher Education Institutions, Suzhou, P.R. China; 4 NAAM Research Group, Faculty of Science, King Abdulaziz University, Jeddah, 21589 Saudi Arabia; 5 Department of Mathematics, Quaid-I-Azam University, Islamabad, 44000 Pakistan

## Abstract

In this paper, biochar derived from poplar catkins was used as an economical and renewable adsorbent for adsorption organic and inorganic pollutants such as, dyes, organic compounds, and heavy metal ions from wastewater. Mesoporous activated carbonized poplar catkins (ACPCs) were produced from char as a by-product by carbonized poplar catkins (CPCs). With their high surface area, ACPCs exhibited the maximum adsorption capacities of 71.85 and 110.17 mg/g for the removal of inorganic U(VI) and Co(II). Compared other biochars adsorbents, ACPCs can also adsorb organic pollutants with the maximum adsorption capacities of 534, 154, 350, 148 and 384 mg/g for methylene blue (MB), methyl orange (MO), Congo red (CR), chloramphenicol (CAP) and naphthalene. The adsorption of organic pollutants was fitted with pseudo-first order, pseudo-second order, and intra-particle diffusion kinetic models figure out the kinetic parameters and adsorption mechanisms. Langmuir adsorption isotherm was found to be suitable for Co(II) and U(VI) adsorption and thermodynamic studies indicated adsorption processes to be endothermic and spontaneous. The adsorption process includes both outer-sphere surface complexes and hydrogen-bonding interactions. The results showed that biochar derived from poplar catkins was a potential material to remove pollutants in wastewater.

## Introduction

Water pollution by organic compounds such as polycyclic aromatic hydrocarbon (PAHs), pesticides, antibiotics and dyes and inorganic heavy metal ions, has been causing considerable worldwide concern^1–3^. Organic pollutants and heavy metal ions management attracts more and more attention of people, because these pollutants are high mobility and long persistence in the environment^4–6^. Therefore, it is significant important to develop an efficient and economical strategy for removal of pollutants in waste water^6–8^.

A number of effective techniques including adsorption, filtration and ion exchange, have been applied to remove inorganic and organic pollutants from waste solutions^9–11^. Among these techniques, adsorption has become one of the most widely used techniques for water pollution management due to its outstanding characteristics, such as low cost, wide adaptability, and convenience^12^. In the past decades, activated carbon has been used to remove pollutants from wastewater but with low regeneration efficiency, which inspire researchers to search for new alternatives with high efficiencies^12–14^. In the quest for economic and effective adsorbents, biomass has been evaluated with variable degree of sources^15, 16^. Low-cost banana and orange peels were carbonized to biochar for removal of dyes from wastewater in Gurusamy Annadurai’s work^17^. Huang *et al*. successfully synthesized biomass adsorbent through the pyrolysis of kapok wadding materials for efficiently solving organic pollution^18^. Physic-chemical characteristics and the adsorption capacity for methylene blue of rice hull ash were systematically studied^19^. Although these biomasses are cheaper than other absorbents, it still suffers some problems, such as remaining economic value, poor adsorption performance and so on.

Poplar alba as a kind of deciduous tree is universally planted in China^20^. Poplar catkins are lightweight and fly around everywhere in summer season. Nevertheless, the flying poplar catkins are harmful for human body in lots of respects, such as promoting runny nose, affecting respiratory system. Moreover, they can cause skin irritation and insomnia^21^. Therefore, it is essential to develop an economically technology for utilizing poplar catkins in large scale. Many reports have been focused on dealing with this problem^20–23^. Though the poplar catkins can be used as biochar, rarely studied has been focused on its application on heavy metal ion and organic pollution.

In this study, carbon micro-tube and mesoporous activated samples were prepared by carbonizing the poplar catkin. The as-prepared samples activated carbonized poplar catkins (ACPCs) and carbonized poplar catkins (CPCs) were characterized by scanning electron microscopy (SEM), transmission electron microscopy (TEM), Fourier transform infrared (FT-IR) spectroscopy, X-ray photoelectron spectroscopy (XPS), Brunauer–Emmett–Teller (BET)-N_2_ surface area and the powder X-ray diffraction analysis (XRD). Besides, the elemental composition was characterized, including C, H, O, and N. The products were then applied adsorption by batch experiments. The possible adsorption mechanism of the pollutants was investigated through FT-IR analysis.

## Results and Discussion

### Characterization of CPCs and ACPCs

**Figure 1:**
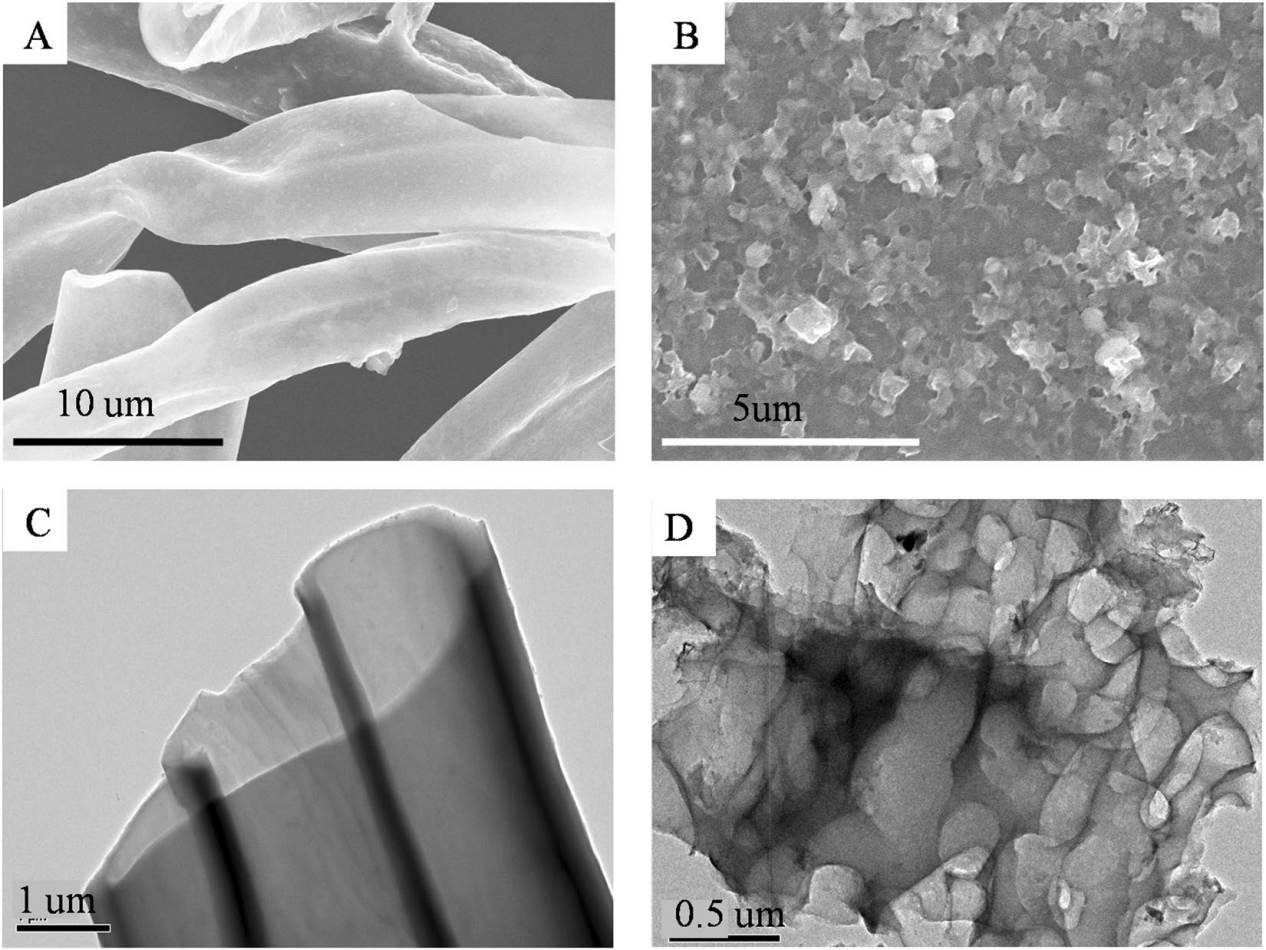
SEM images for CPCs (**A**) and ACPCs (**B**) TEM for CPCs (**C**) and ACPCs (**D**).

The morphologies of CPCs and ACPCs were characterized by using SEM and TEM analysis. The CPCs samples showed hollow micro-tubular structure with the thin layers^24, 25^ (Fig. 1A) while the SEM image of ACPCs (Fig. 1B) displayed a network and porous structure stacked by irregular flakes, which could be considered as high specific surface area. The TEM image of CPCs (Fig. 1C) confirmed its hollow micro-tubular structure while ACPCs (Fig. 1D) were network structure stacked by irregular flakes. The structure of ACPCs facilitated the rapid adsorption process by accelerating the transportation of the pollutants from the outer surface into the inner porous network^25^.

**Figure 2:**
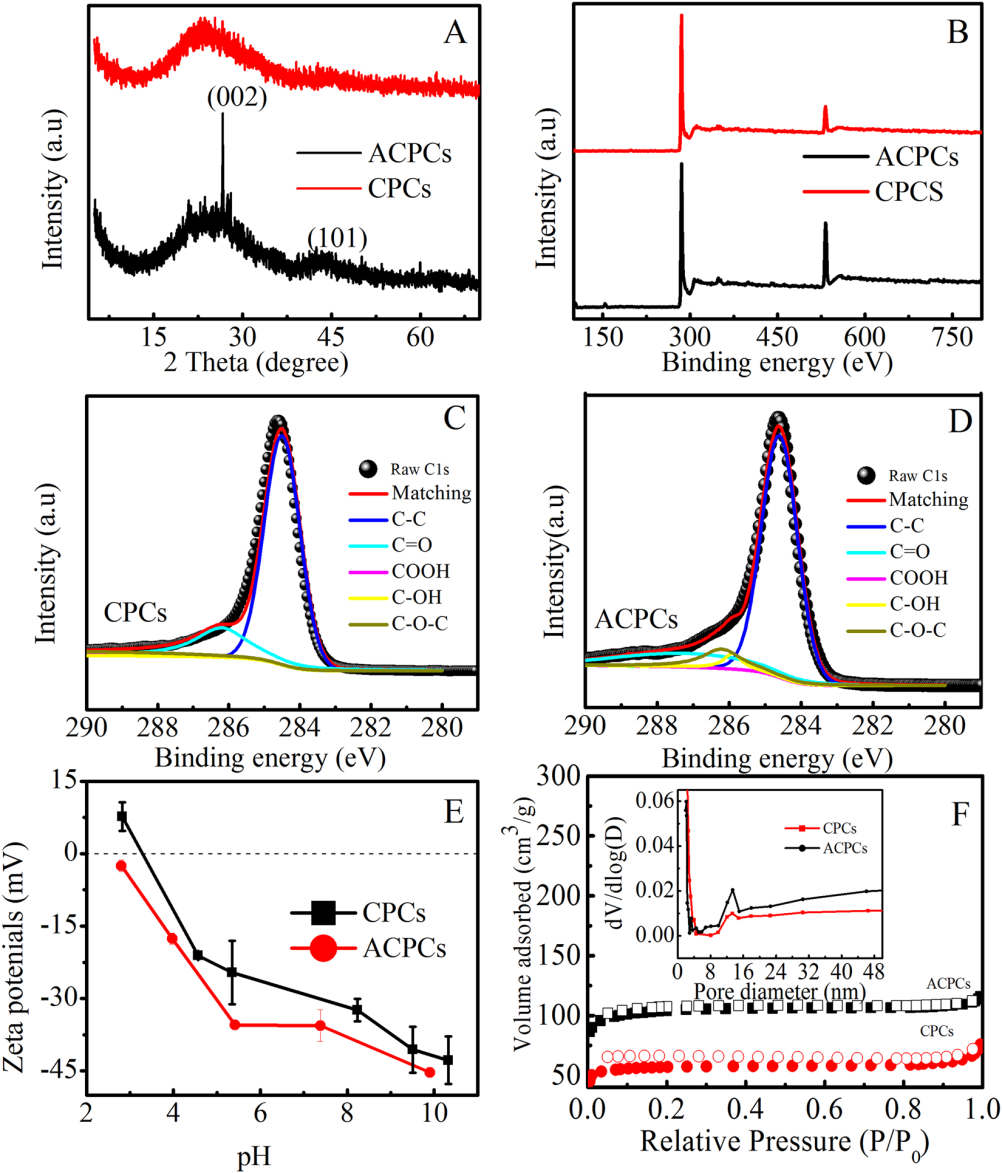
XRD patterns of CPCs and ACPCs (**A**) XPS spectrums of CPCs and ACPCs. (**B**) C1s XPS spectrums of CPCs (**C**) and ACPCs (**D**) Zeta potentials of CPCs and ACPCs at various pH values (**E**) Nitrogen adsorption–desorption isotherms of CPCs and ACPCs (**F**).

XRD patterns of the CPCs and the ACPCs were shown in Fig. 2A. The as-prepared ACPCs displayed a broad feature at 24° and a weak one at 43°, corresponding to the (002) and (101) planes, respectively, demonstrating amorphous structures. As compared with ACPCs, the peak at 43^o^ was weaker in CPCs, indicating a lower degree of interlayer condensation in CPCs^26–29^. The XPS results of CPCs and ACPCs applying to detect the surface states were shown in Fig. 2B. Strong C 1s peaks were observed in both spectra, indicating dominant carbon element in both adsorbents. However, the ratio of C/O increased from 3.67 to 7.73 for CPCs and ACPCs, indicating an increased oxygen degree after KOH treatment^29^. The C 1s peaks of CPCs (Fig. 2C) and ACPCs (Fig. 2D) were further resolved into five peaks, i.e. C-C, C-OH, C-O-C, C=O, and C-OOH with binding energies of 284.5, 286.2, 287.1, 288.0, and 289.5 eV, respectively^30^. The elemental composition, including C, H, O, and N, are shown in Table S1. The relative amount of C element decreased from about 64% to 58% after KOH treatment, suggesting an increased oxygen degree after KOH treatment.

Zeta potentials were used to analyze surface properties of CPCs and ACPCs in aqueous solution (Fig. 2E). The pH_pzc_ values (point zero of charge) of CPCs and ACPCs, mainly determined by its chemical nature^31^, were estimated to be 3.5 and 3.0, respectively. ACPCs had a much lower zeta potential than CPCs, suggesting a more negatively charged surface of ACPCs in water than that of CPCs, which can be caused by oxygen-abundant functional groups on the surface of ACPCs^32^. The more negatively charged surface led to adsorb positively charged pollutant more easily and more oxygen-abundant functional groups resulted in chemisorptions more obvious^33^. The BET surface area, an important influencing factor for adsorption capacity, gave values on the specific surface area and pore structure of ACPCs and CPCs. Figure 2F was obtained from N_2_ adsorption–desorption isotherm measurements. The larger surface areas (351.4 and 191.1 m^2^/g for ACPCs and CPCs) and slightly smaller average pore diameter (2.15 and 1.96 nm for CPCs and ACPCs) was achieved, indicating better development of the porosity after KOH activation. These values were also higher than many reported biochars as listed in Table S2.

### Adsorption study

#### Organic dyes adsorption

**Figure 3:**
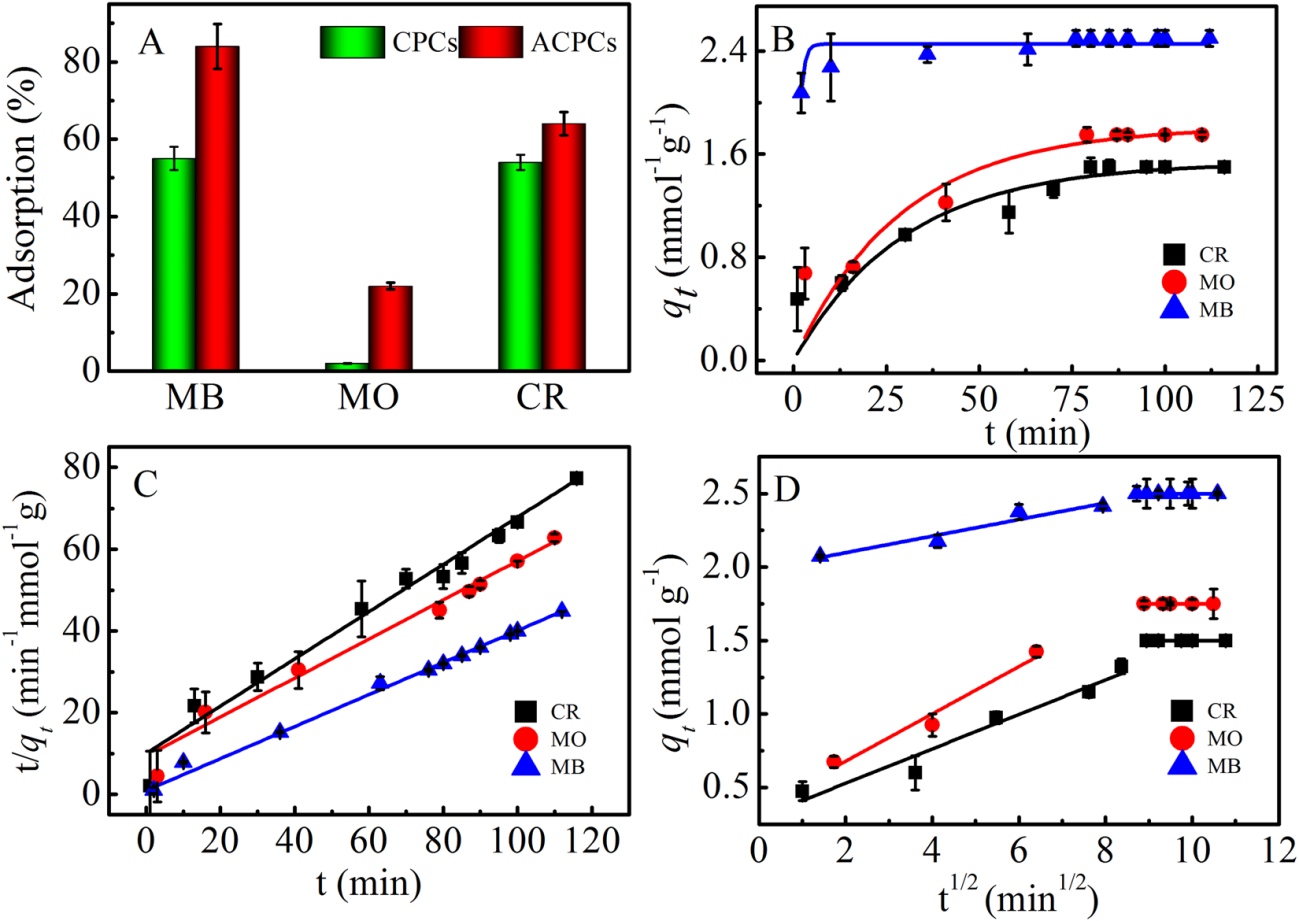
Adsorption capacities toward MB, MO and CR of ACPCs and CPCs (**A**) pseudo-first-order (**B**) pseudo-second-order (**C**) intraparticle diffusion (**D**) C_0_ = 0.016 mM, m/V = 0.04 g L^−1^, T = 303 K, pH = 6.0.

The adsorption capacities of ACPCs and CPCs with positively charged methylene blue (MB), negatively charged methyl orange (MO) and neutral Congo red (CR) were studied and depicted in Fig. 3A. ACPCs exhibited the highest adsorption capacity toward MB, which is 1.5 times higher than that of CPCs. The adsorption capacity was got from adsorption isotherms shown in Figure S1. The adsorption performances (%) of ACPCs toward MO and CR were 14 and 22%, which 10 times and 1.2 times than that of CPCs. By comparing the adsorption data, the adsorption capacity toward MB was better than both MO and CR, which could be ascribed to the negative zeta potentials of ACPCs and CPCs^33, 34^. MB surface was positively charged, MO was negatively charged and while CR was neutral. Therefore, the electrostatic attraction force for MB was stronger than MO and CR. The adsorption performances of ACPCs toward organic dyes (MB, MO and CR) were better than CPCs, which attributed to oxygen-containing functional groups and their surface areas. The results were consistent with many reports^2, 7, 8, 31, 35^. For example, active carbon displayed much higher adsorption capacity toward MB than graphene oxide and carbon nanotubes which was related with its highest surface area in Li’s work^35^. Chen *et al*. reported that cotton derived porous carbon oxide possessed the advantage of a higher adsorption capacity than cotton derived porous carbon, which confirmed that oxygen-containing functional groups could enhance adsorption^31^. Table 1 presented comparative adsorption capacities of reported biochars. Adsorption capacities of ACPCs were higher than many other biochars. Thus, the comparison of adsorption capacities showed that ACPCs was an efficient adsorbent for the uptake of dyes. It was worth noting that there was still some biochars having higher adsorption capacity than ACPCs, whose functional groups could greatly enhance adsorption capacity.

**Table 1:** Comparison of various adsorbent materials.

Adsorbents	Adsorbed substances	*q*_*e*_(mg/g)	Ref
Banana peel	MO	21	36
Tobacco stem ash	MB	35.7	49
Banana peel	CR	18.2	36
Orange peel	MO	20.5	36
Orange peel	MB	18.6	36
Orange peel	CR	14.3	36
Apricot shell	MB	4.11	49
wheat straw	MO	278.7	50
Rice husk	MB	578	2
waste polystyrene	CR	500	51
Poplar catkins	MB	534	This work
	MO	154	
	CR	350	
Bamboo charcoal	CAP	8.1	56
Crop residue	CAP	742.4	57
Bean pods	naphthalene	300	58
Orange peels	naphthalene	80.6	1
Poplar catkins	CAP	148	This work
	naphthalene	384	
Penicillium citrinum	U(VI)	255.1	59
Rice husk	Co(II)	45.2	60
Almond shell	U(VI)	28.09	61
Seaweeds	Co(II)	18.58	62
Poplar catkins	U(VI)	71.85	This work
	Co(II)	110.17	

Adsorption kinetics, an important adsorbent design factor, can directly reflect the sorbent uptake rate. In this study, the adsorption kinetics were conducted with the sample concentration of 0.04 g·L^−1^ at 303 K. Three kinetic models were used to analyze the mechanism of the adsorption process, i.e. pseudo-first order model (Fig. 3B), pseudo-second order model (Fig. 3C), and intra-diffusion (Fig. 3D) and the related parameters are in Table 2.

**Table 2:** Parameters for Kinetics Models for the Adsorption of MB, MO, and CR.

Adsorbate	pseudo-first-order		pseudo-second-order		Intra-diffusion model	
MB	k_1_	0.033	k_2_	0.015	k_3_	0.079
	*q*_*e*_	0.409	*q*_*e*_	0.42	C_3_	0.33
	R^2^	0.85	R^2^	0.998	R^2^	0.96
MO	k_1_	0.95	k_2_	0.024	k_3_	0.115
	*q*_*e*_	0.302	*q*_*e*_	0.35	C_3_	0.29
	R^2^	0.88	R^2^	0.995	R^2^	0.98
CR	k_1_	0.033	k_2_	0.36	k_3_	0.012
	*q*_*e*_	0.256	*q*_*e*_	0.29	C_3_	0.25
	R^2^	0.89	R^2^	0.997	R^2^	0.97

The pseudo first-order equation describes adsorption in solid–liquid systems. It expressed as following^36–38^:

1 $$\mathrm{log}({q}_{e}-{q}_{t})=\,\mathrm{log}\,{q}_{e}-\frac{{k}_{1}}{2.303}t$$

where *q*_*e*_ and *q*_*t*_ (mmol g^−1^) are the adsorption capacities at equilibrium and at time *t* (min), respectively.

The pseudo second-order rate expression, which was applied for analyzing chemisorption kinetics from liquid solutions, was linearly expressed as^39^:

2 $$\frac{{\rm{t}}}{{q}_{t}}=\frac{1}{{k}_{2}{q}_{e}^{2}}+\frac{1}{{q}_{e}}t$$

where *k*_2_ is the rate constant for pseudo second-order adsorption (g mmol^−1^ min^−1^).

By comparing the correlation coefficients (R^2^), pseudo-second order kinetic model (0.995–0.998) exhibited much higher correlation coefficients than pseudo-first order kinetic model (0.85–0.89), demonstrating a pseudo-second order kinetic model for the adsorption of MB, MO, and CR onto ACPCs. However, the investigation of diffusion mechanisms and rate controlling procedure were beyond the scope of pseudo-first and pseudo-second order kinetic models, intra-particle diffusion model was applied for further investigation.

Intraparticle diffusion focuses on the adsorbate transportation from external surface to pores and the rate-controlling adsorption process, respectively. The model could be expressed as^40^:

3 $${q}_{t}={k}_{i}{t}^{1/2}+C$$

where k_i_ is the diffusion rate constant and C is a constant.

As shown in Fig. 3D, the plot of q_t_ against t^1/2^ includes two linear sections, which correspond to a two-step adsorption process. The first linear line indicated the diffusion of dyes from solutions to the surfaces of ACPCs, followed by a slow diffusion of dyes into pores. The second linear section with a smaller slope indicated the final equilibrium stage where the intra-particle diffusion started to slow down and to reach the final equilibrium status.

### Chloramphenicol (CAP) and naphthalene adsorption

**Figure 4:**
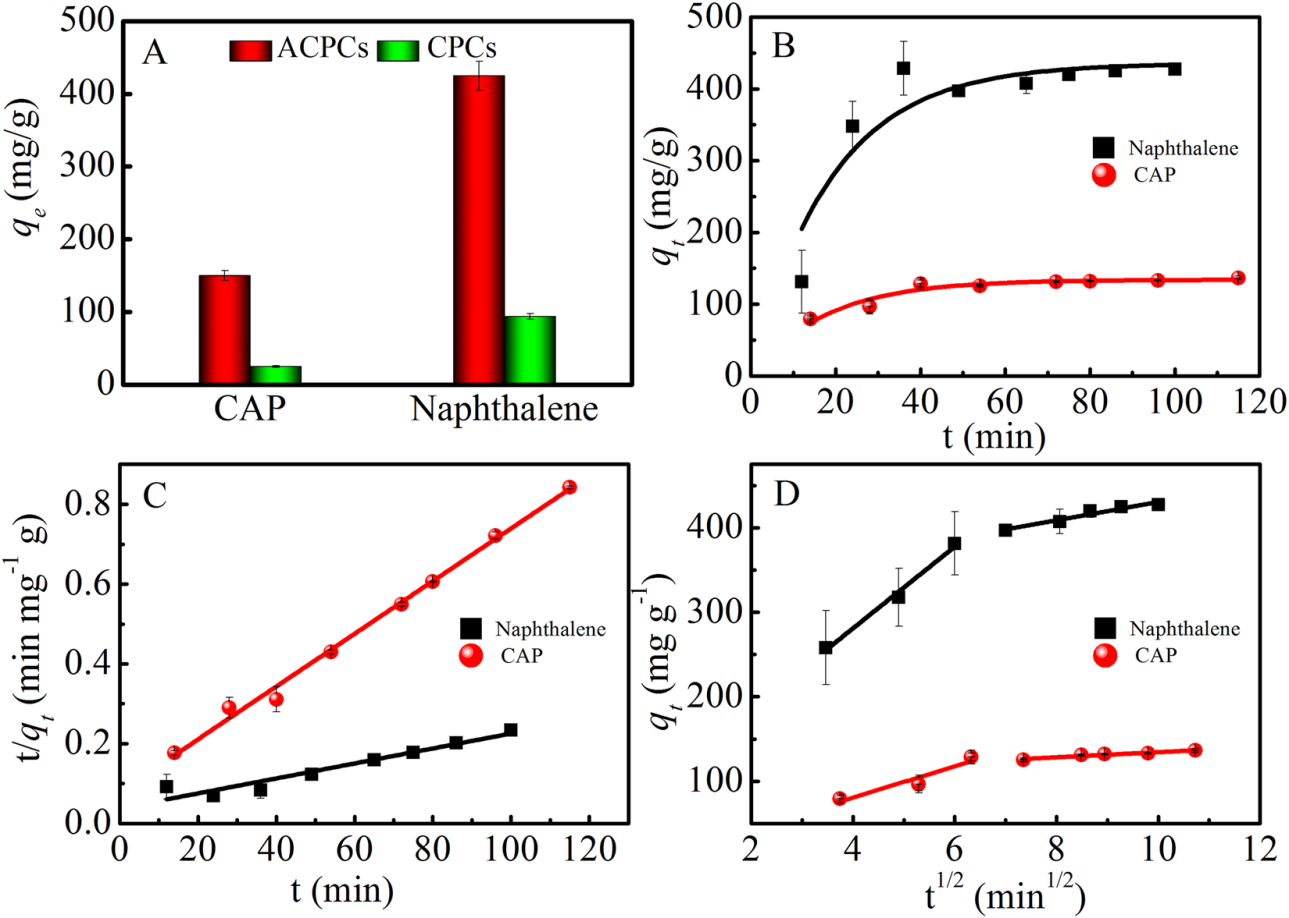
Adsorption capacities toward CAP and naphthalene of ACPCs and CPCs (**A**) pseudo-first-order (**B**) pseudo-second-order (**C**) intraparticle diffusion (**D**) C_0_ = 30 mg/L, m/V = 0.04 g L^−1^, T = 303 K, pH = 7.0, I = 0.01 mol L^−1^ NaNO_3_.

Figure 4A depicted adsorption capacity of 30 mg L^−1^ of CAP and naphthalene, respectively by 0.04 g L^−1^ of ACPCs and CPCs for 12 hours at pH 7.0. A higher adsorption capacity of ACPCs than CPCs for organic pollutant can be found. The results of the CAP and naphthalene kinetics data fitted to the various theoretical models were shown on Fig. 4B–D. The corresponding kinetic parameters calculated from the different equations are compiled in Table S3. Pseudo-first-order and intra-particles models suggested that they may not be suitable to describe the kinetics of naphthalene adsorption on ACPCs. On the contrary, R^2^ of the pseudo-second-order model is higher than other two models, which indicated that the pseudo-second order kinetic model can be can well describe the adsorption process^41, 42^.

### Heavy metal ions adsorption

**Figure 5:**
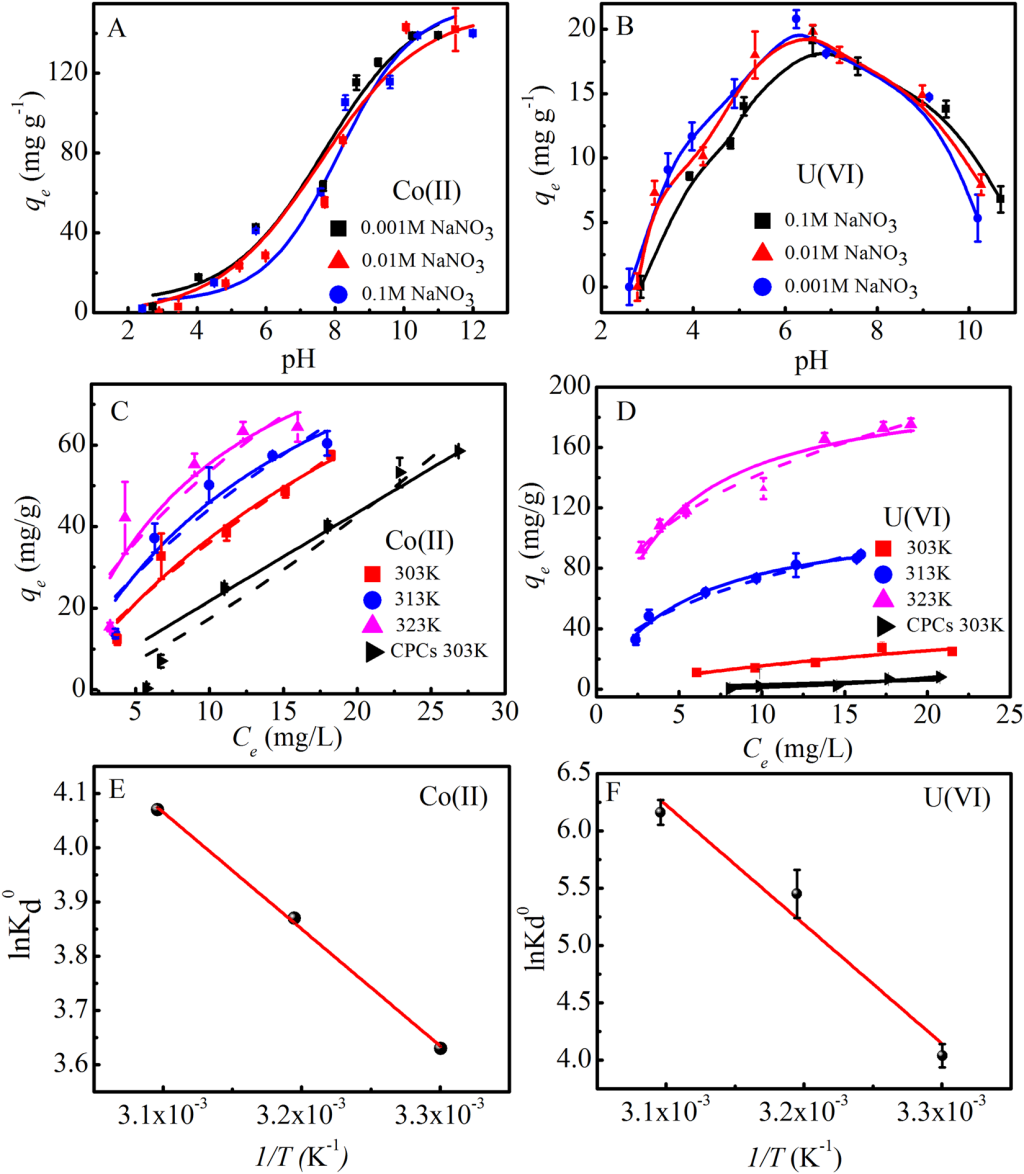
Effect of pH and ionic strength on the adsorption of (**A**) Co(II) and (**B**) U(VI) onto ACPCs; Adsorption isotherms of Co(II) and U(VI) on ACPCs at different temperatures and CPCs at 303 K. Co(II): pH = 6.0, m/V = 0.1 g L^−1^, I = 0.01 mol L^−1^ NaNO_3_ (**C**) U(VI): pH = 5.0, m/V = 0.1 g L^−1^, I = 0.01 mol L^−1^ NaNO_3_ (**D**) Plots of lnk_d_^0^ vs. 1/T for Co(II) adsorption (**E**) and for U(VI) adsorption (**F**) on ACPCs C_0_ = 15.0 mg L^−1^, m/V = 0.1 g L^−1^, T = 303 K.

The adsorption capacities of ACPCs toward inorganic Co(II) and U(VI) were also investigated. The adsorption of Co(II) on the ACPCs was highly dependent on pH (Fig. 5A). The Co(II) adsorption on ACPCs increased slowly at pH 2.0 to 5.0, then increased sharply within pH 5.0 to 10.0, and reached the maximum at pH > 10.0^43^. This phenomenon may be contributed to the electrostatic attraction between Co(II) and ACPCs^43, 44^. Cobalt existed in the form of Co^2+^, Co(OH)^+^, Co(OH)_2_, and Co(OH)_3_^−^ at different pH values (Figure S2A). Low adsorption efficiency of Co(II) at pH < 5.0 on ACPCs was ascribed to the competition with H^+^ ions for the binding sites onto ACPCs^45, 46^. The surface deprotonation reaction at high pH values resulted in deprotonated sites increased with increasing pH. The deprotonated sites were easier to keep the Co(II) ions, and surface complexation between Co^2+^, Co(OH)^+^, and ACPCs is strengthen, thus the Co(II) adsorption capacity increase sharply at pH 6.0–8.5 and Co(OH)_2_ precipitation begins to form at pH > 8.5.

The influence of ionic strength toward Co(II) adsorption was also examined within the tested pH ranges (Fig. 5A). The ionic strength affected the thickness of the double layer, and resulted the binding ability towards the adsorbing species^47^. The ionic strength-dependent adsorption capacity suggested that electrostatic outer-sphere complexation reactions might be a sorption mechanism for Co(II) adsorption.

As shown in Fig. 5B, the adsorption of U(VI) on ACPCs surface increased with increasing pH from 2.5 to 5.0, then reached a maximum adsorption capacity of about 20 mg/g at pH 6.0, while decreased adsorption was observed at pH > 6.5. The species distribution of U(VI) at different pH values can be used to explain the observed adsorption tendency as shown in Figure S2B. The major species of U(VI) in aqueous was UO_2_^2+^ at pH < 5.0, and more multiple positive charged U(VI) species were observed at pH 5.0–8.0, whereas the negative charged U(VI) species were observed at pH > 8.0^45, 47^. Influence of ionic strength on the adsorption of U(VI) was studied in different NaNO_3_ solutions. It can be seen that ionic strength has major effect on the U(VI) adsorption, suggesting that an outer-sphere surface complexation dominant adsorption process^48, 49^.

### Adsorption Isotherms

The adsorption isotherms for Co(II) and U(VI) obtained at 303, 313, and 323 K were shown in Fig. 5C and D. To have a better understanding of the mechanism and to quantify the adsorption data, Langmuir and Freundlich isotherm models were conducted to simulate the adsorption. The Langmuir isotherm model $({q}_{e}=\frac{b{q}_{\max }{C}_{e}}{1+b{C}_{e}})$ describes monolayer adsorption process^50, 51^. *q*_*max*_ (mg/g) represented the maximum adsorption capacity, and b (L/mg) is the constant that corresponds to the heat of adsorption. The Freundlich isotherm model, which allowed for several kinds of adsorption sites on the solid, can be represented by the equation (${{\rm{q}}}_{{\rm{e}}}={K}_{F}{C}_{e}^{n}$)^52^, where *K*_*F*_ (mg^1−*n*^ L^n^/g) represented the adsorption capacity when the adsorbate equilibrium concentration was equal to 1, and n expressed the degree of dependence of adsorption with equilibrium concentration.

Langmuir and Freundlich isotherms parameters for Co(II) and U(VI) adsorption on ACPCs were shown in Table 3. Higher correlation coefficients were observed for the Freundlich model than that of the Langmuir mode, suggesting multiple adsorption sites on the ACPCs surfaces to achieve adsorption process.

**Table 3:** Langmuir and Freundlich isotherms parameters for Co(II) and U(VI) adsorption on ACPCs.

M	T (K)	Langmuir model			Freundlich model		
		*q*_max_(*mg*/*g*)	*b*(*L*/*g*)	R^2^	*K*_*F*_ (*mg* ^*1*−*n*^ *L* ^*n*^/*g*)	n	R^2^
Co(II)	303	110.17	0.032	0.93	6.21	0.76	0.95
	313	121.85	0.06	0.94	9.80	0.65	0.95
	323	152.25	0.10	0.98	14.5	0.56	0.96
	303	71.85	0.02	0.94	2.82	0.73	0.95
U(VI)	313	116.82	0.18	0.95	26.99	0.43	0.96
	323	204.09	0.27	0.98	67.19	0.32	0.97

Thermodynamic parameters (ΔG°, ΔS° and ΔH°) of the Co(II) and U(VI) adsorption on ACPCs calculated from isotherms on three different temperature, were investigated to determine whether the adsorption process occurred spontaneously. The value of the Gibbs free energy change (ΔG°) could be achieved by the following equation^52^:

4 $${{\rm{\Delta }}H}^{0}={{\rm{\Delta }}G}^{0}+{T{\rm{\Delta }}S}^{0}$$

where R (8.314 J/mol K) was the ideal gas constant and T was the temperature in Kelvin. The adsorption equilibrium constant *K*_0_ can be calculated by plotting ln*K*_d_ versus *C*_e_ and extrapolating *C*_e_ to zero.

The average standard enthalpy change (*ΔH* ^*0*^) and standard entropy change (*ΔS* ^*0*^) could be calculated from the slope and y-intercept to plot of ln*K*_*d*_^*0*^ versus 1/T (Fig. 5E and F) using the Van’t Hoff equation^5, 53^:

5 $$\mathrm{ln}\,{K}_{{\rm{d}}}^{0}=\frac{{\rm{\Delta }}{S}^{0}}{R}-{\frac{{\rm{\Delta }}H}{RT}}^{0}$$

where *K*_*d*_ was the adsorption equilibrium constant. Table 4 listed ΔG^0^, ΔS^0^ and ΔH^0^ calculated from the adsorption isotherms at three different temperatures. The adsorption process can be considered as a positive values of *ΔH* ^0^ indicated an endothermic (positive values of *ΔH* ^0^) and spontaneous (negative values of *ΔG* ^0^) adsorption process.

**Table 4:** Thermodynamic parameters for the adsorption of Co(II) and U(VI) by ACPCs.

	T (K)	**Δ** *G* ^0^(kJ/mol)	**Δ** *H* ^0^(kJ/mol)	**Δ** *S* ^0^(J/mol/K)
Co(II)	303	−9.03	17.9	88.9
	313	−9.92		
	323	−10.8		
U(VI)	303	−10.4	86.5	319.9
	313	−13.5		
	323	−16.8		

### Mechanism of Adsorption

**Figure 6:**
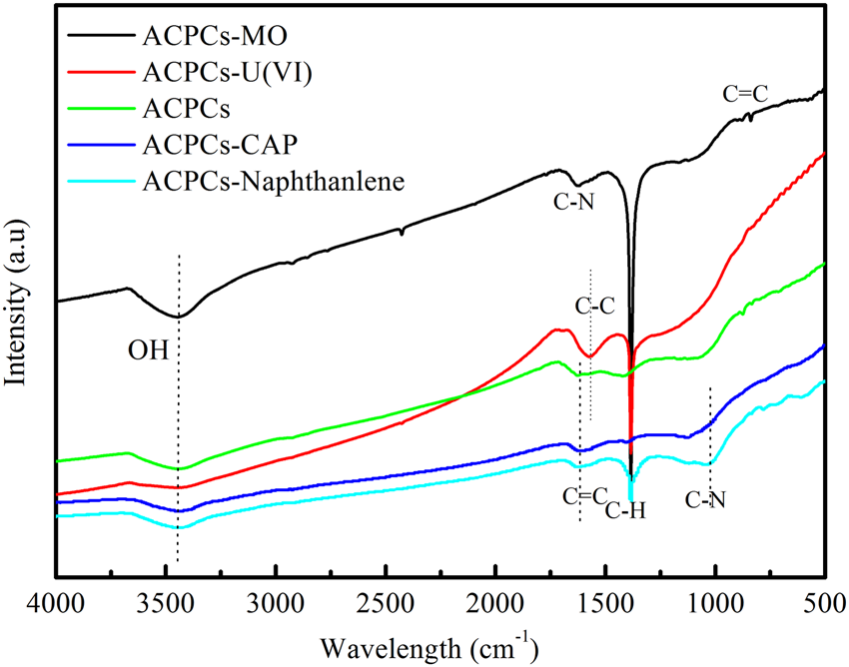
FTIR spectra of ACPCs after adsorption pollutants.

FT-IR spectra were measured to investigate the adsorption mechanism (Fig. 6). One typical dye and ions were displayed here and others were shown in Figure S3. IR spectrum of all results showed peaks at 3450 cm^−1^ which can be assigned to the O-H stretching vibration mode of hydroxyl functional groups. The obvious peaks around 1660–1550 cm^−1^ at ACPCs after adsorption heavy metal ions (Co(II)/U(VI)) and organic pollution because of the presence of highly conjugated C-O. An obvious peak at 2903 cm^−1^ after adsorption organic dyes (MB, MO and CR) clearly indicated the presence of aliphatic C-H stretching from organic dyes^54^. Similarly, the overlapping bands in the region of 800–600 cm^−1^ after organic dye adsorption may be ascribed to the out of plane ring deformation of organic dyes^55^. The results clearly indicated chemisorption was in the process. In the region of 1030–1200 cm^−1^ after CAP and naphthalene adsorption may be C-N in CAP and naphthalene which indicated successfully sorbed CAP and naphthalene.

In conclusion, CPCs and ACPCs were successfully fabricated from poplar catkin. The prepared biochars exhibited good to excellent adsorption capacities for both organic pollutant (MB, MO, CR, CAP and naphthalene) and heavy metal ions (Co(II) and U(VI)). The adsorption of U(VI) and Co(II) from aqueous solutions onto ACPCs were highly dependent on solution pH and ionic strength, indicating outer-sphere surface complexation dominated the adsorption mechanism. Thermodynamic studies indicated endothermic and spontaneous processes. Kinetic results showed that pseudo-second-order model was better fitted in the removal of dyes process. FT-IR results indicated the participation of the specific functional groups in adsorption interaction. The biochars derived from poplar catkins showed great potential in wastewater management in terms of their facile process, low cost and good ability of adsorption of heavy metals and organic pollution.

## Experimental Section

### Chemicals

Analytical pure reagent naphthalene and dyes were purchased from Sinopharm Chemical Reagent Co., Ltd. CAP was purchased from Aladdin Industrial Corporation (Shanghai, China). Properties of the organic pollutants adsorbates are listed in Table S4. The Co(II) and U(VI) stock solutions (60 mg/L) were prepared by dissolving their nitrate in Milli-Q water. The synthetic naphthalene aqueous solutions were prepared from a stock solution (60 mg/L) in ethanol (less than 5 wt. %) by adequate dilution in Milli-Q water because of the low water solubility of naphthalene (30 mg/L).

### Synthesis of adsorbents

**Figure 7:**
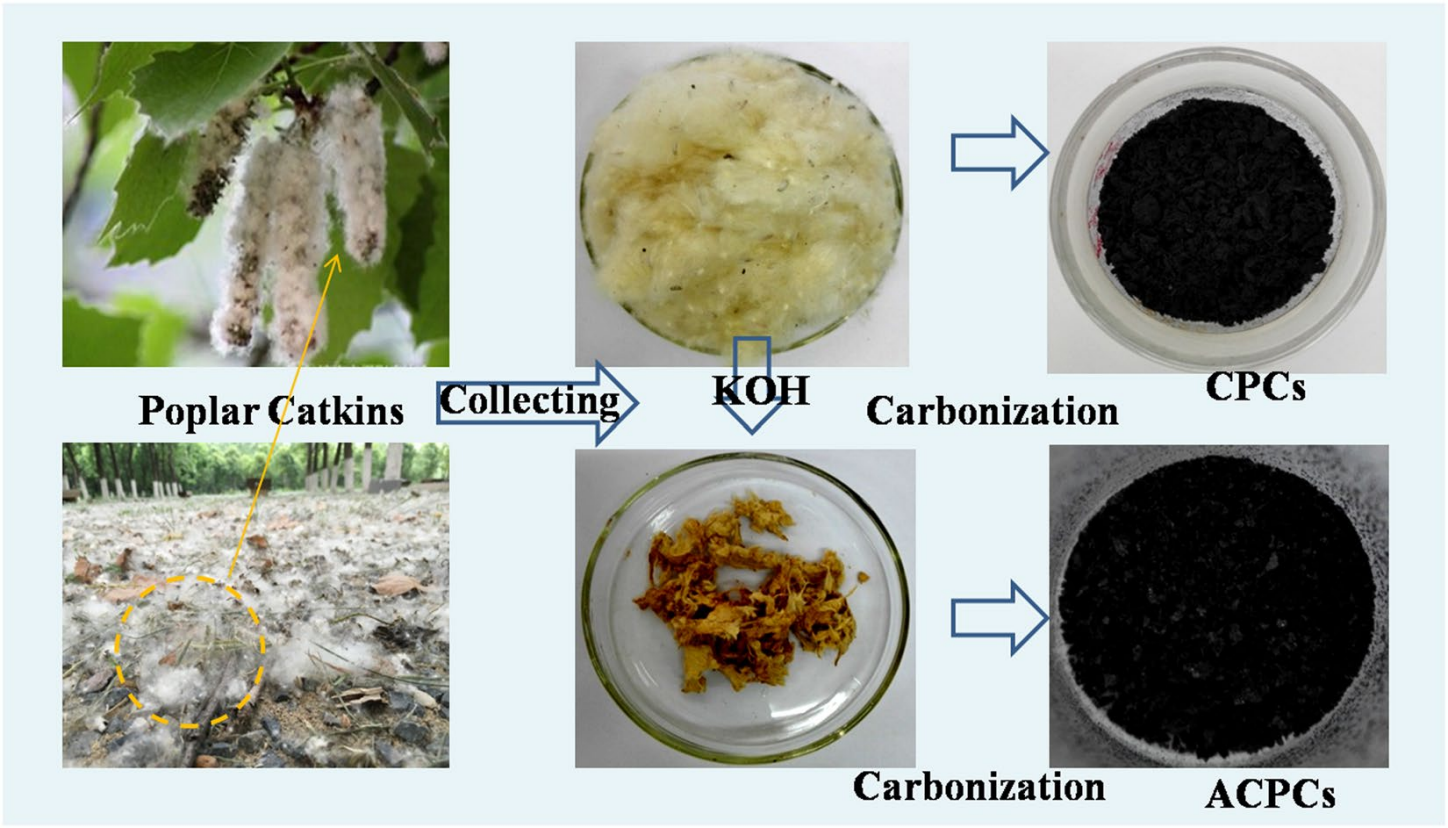
Detailed flow chart of the process for CPCs and ACPCs from poplar catkins.

The poplar catkins were collected in Shushan in Hefei, Anhui province in China (Fig. 7). The fabrication of CPCs and ACPCs were listed as follows: Specifically, 2.0 g of the collected poplar catkin was washed with deionized water to remove the impurity and dried at 70 °C overnight. Half of dried poplar catkin (~1.0 g) was dispersed in 40 mL 3.0 mol/L KOH solution with ultra-sonicating for 30 min, and the mixture were dried at 65 °C for 24 h, followed by carbonization under nitrogen atmosphere by heating at 800 °C for 1 h with a heating rate of 1 °C min^−1^ to produced desired ACPCs. CPCs were obtained of the other half dried poplar catkin (~1.0 g) by directly carbonization under same condition with ACPCs. The detailed fabrication process was schematically shown in Fig. 7. An optimization study aimed at identifying preferred conditions (carbonization/activation) for preparing ACPCs was given in supporting information.

### Characterization

SEM images were taken by a JEOL JSM-7001F microscope. TEM images were obtained using a JEOL-2010 transmission electron microscope with an accelerating voltage of 200 kV. XRD were taken on a Philips X’Pert X-ray diffractometer using Cu Kα radiation (λ = 0.15406 nm). XPS spectra were obtained on an AXIS Ultra DLD spectrometer with an excitation source of Mg Kα (1486.6 eV). FT-IR spectroscopy was performed using a Nicolet Magna-IR 750 spectrometer over a wave number range from 400 to 4000 cm^−1^. The zeta potentials were measured using a Zetasizer Nanosizer ZS instrument (Malvern Instrument Co., UK). Elemental composition was characterization by Vario EL cube, Elementar, Germany. Ash content of the samples was determined by combusting 0.5 g of adsorbent in a muffle furnace at 650 °C for 2 h.

### Batch adsorption experiments

Adsorption experiments of heavy metal ions were performed in polyethylene test tubes and adsorption experiments of organic pollutants were conducted in 10 mL glass bottle by using batch experiment. The pH of the suspension in the range of 2.0–10.0 was adjusted by adding negligible amount of 0.1 or 0.01 mol/L HNO_3_ or NaOH solutions. The suspensions were then agitated on a shaker for a reaction time of 24 h. The solid phase was separated from the solution phase by centrifugation at 8000 rpm for 10 min.

The concentrations of Co(II) were determined by a spectrophotometric method using Co(II)-Xylenol orange complex at the wavelength of 578 nm and the concentration of U(VI) was analyzed by the Dichlorophosphonoazo III spectrophotometer method at the wavelength of 669 nm. The concentration of organic pollutant was analyzed by UV-vis (Shimadzu UV-2550) spectroscopy (the maximum absorption wavelength for every pollutant was shown in Table S3). The adsorption percentage (%) and adsorption capacity (*q*_*e*_) which were achieved by the following equations^5, 23^:

6 $$Adsorption \% =\frac{{C}_{0}-{C}_{e}}{{C}_{0}}\times 100 \% $$

7 $${q}_{e}=\frac{{C}_{0}-{C}_{e}}{m}\times V$$

where *C*_0_ (mg/L) is initial concentration and *C*_e_ (mg/L) is final concentration of pollution in the aqueous phase, m (g) is mass of the adsorbent, and V (mL) is the volume adsorption solution. All the experimental data were the averages of duplicate determinations. Error bars represent standard deviation.

## Electronic supplementary material

Supplementary information
