# Supplementary material for: A Valuable Biochar from Poplar Catkins with High Adsorption Capacity for Both Organic Pollutants and Inorganic Heavy Metal Ions
Source: Sci Rep. 2017 Aug 30;7:10033. doi: 10.1038/s41598-017-09446-0 (PMC5577165; doi:10.1038/s41598-017-09446-0)
Supplement: Supplementary file 1 — Supplementary information [file 41598_2017_9446_MOESM1_ESM.doc]

**Supporting information for**

**A Valuable Biochar from Poplar Catkins with High Adsorption Capacity for** **Both** **Organic Pollutants and Inorganic Heavy Metal Ions**

**Xia Liu****^1,2^, Ju Sun^1,2^, Shengxia Duan^1,2^, Yanan Wang^1,2^, Tasawar Hayat^4^,** **Ahmed Alsaedi, ^4,5^ Chengming Wang^2^& Jiaxing Li^1,3,4^**

^1^ Institute of Plasma Physics, Chinese Academy of Sciences, P.O. Box 1126, Hefei, 230031, P.R. China

^2^ University of Science and Technology of China, Hefei, 230026, P.R. China

^3^ Collaborative Innovation Center of Radiation Medicine of Jiangsu Higher Education Institutions, P.R. China

^4^ NAAM Research Group, Faculty of Science, King Abdulaziz University, Jeddah 21589, Saudi Arabia.

^5^ Department of Mathematics, Quaid-I-Azam University, Islamabad 44000, Pakistan.

*Corresponding author. Tel.: +86-551-65596617; Email: lijx@ipp.ac.cn (J. Li)


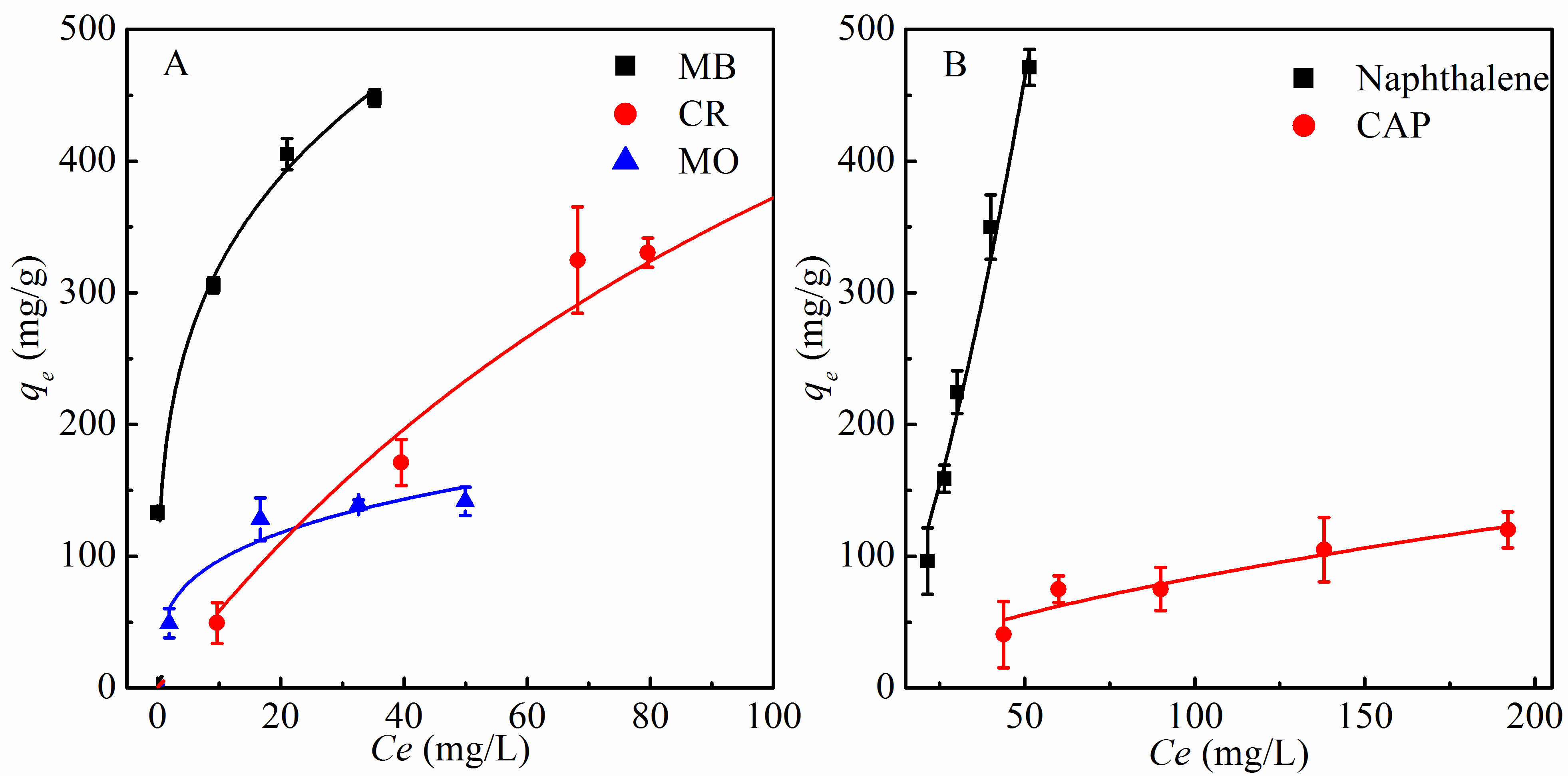


Figure S1 The adsorption isotherms of MB, MO and CR (A) naphthalene and CAP to ACPCs from aqueous solution (B) m/V=0.04 g L^-1^, T=303 K, pH=7.0, C_NaNO3_=0.01M


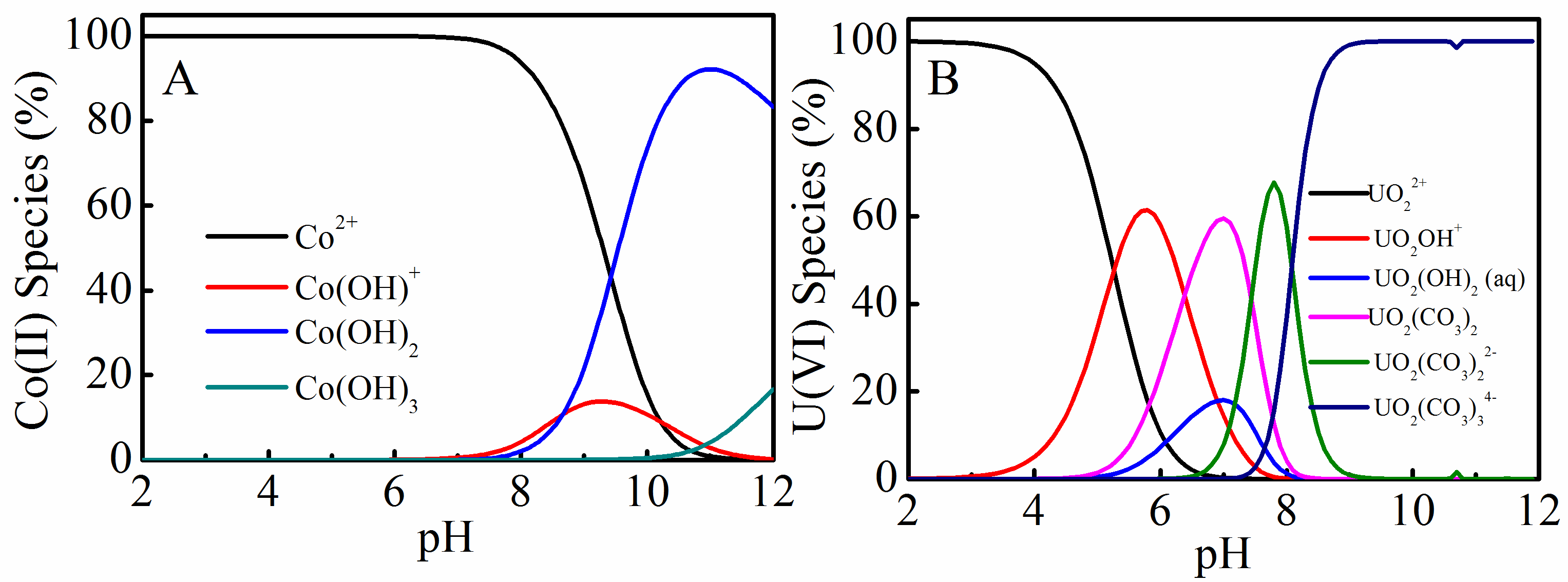


Figure S2 The species of Co(II) (A) and U(VI) (B) at various pH by Visual Minteq 3.1.


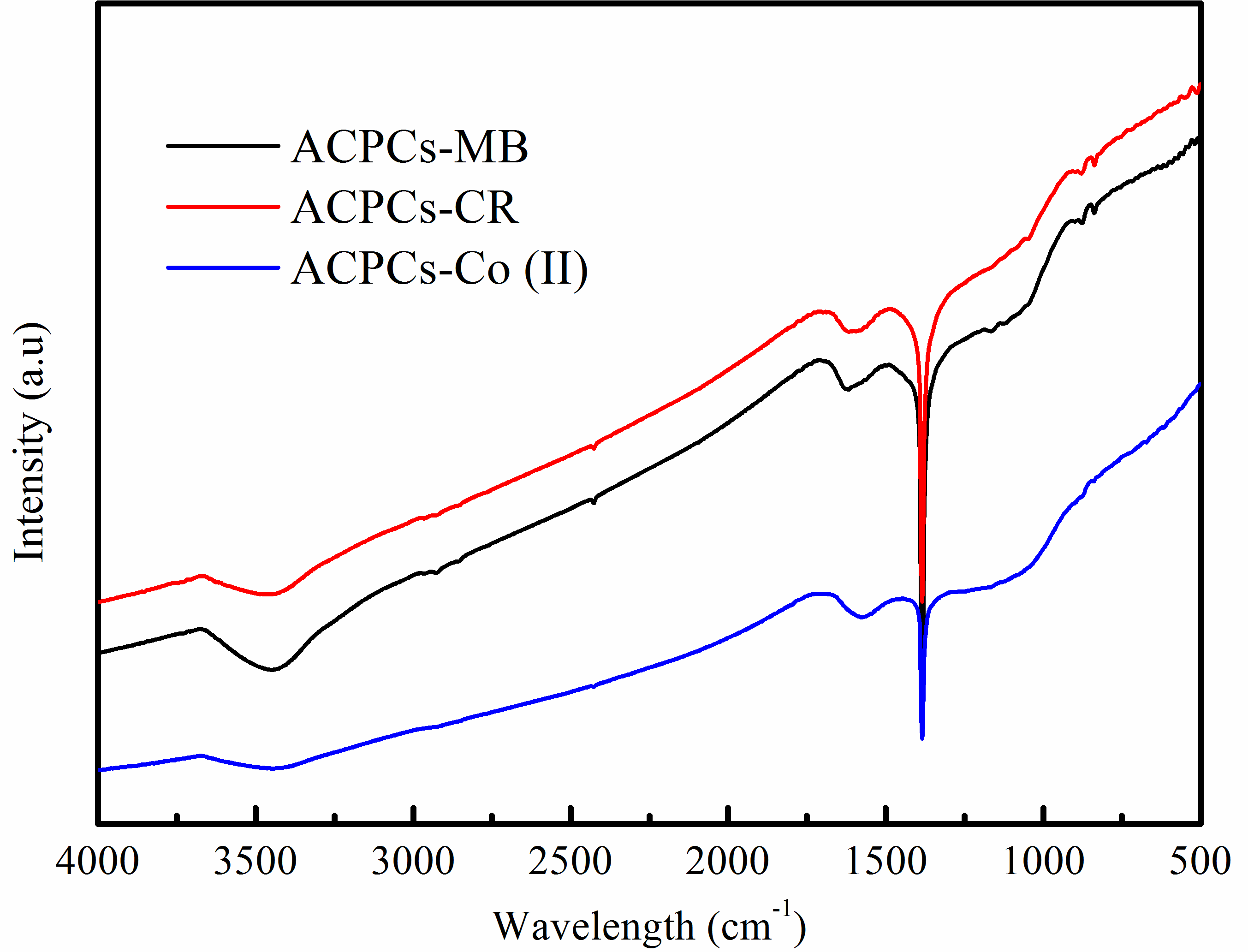


Figure S3 FTIR for ACPCs after adsorption MB, CR and Co(II)

**Optimization Experiment.** An optimization study aimed at identifying preferred carbonization/activation conditions for preparing ACPCs was done. Dried poplar catkin (~1.0 g) was dispersed in 40 mL 3.0 mol/L KOH solution with ultra-sonicating for 30 min, and the mixture were dried at 65 ^o^C for 24 h, followed by carbonization under nitrogen atmosphere by heating at 600, 700 and 800 ^o^C for 1 h with a heating rate of 1 ^o^C min^-1^ to produce desired ACPCs-600, ACPC-700, ACPC-800. The XRD patterns for ACPCs-600, ACPC-700, ACPC-800 (Figure S4). Two broad peaks located at approximately 24^o^ and 43^o^ can be attributed to the (0 0 2) and (1 0 0) reflections of the disordered carbon layer. The high intensity in the low angle region indicates the existence of abundant micropores in the samples. Furthermore, with increasing the carbonization temperature from 600 to 800 ^o^C, the intensity of (1 0 0) peak was gradually enhanced, implying the improvement of the graphitization degree of ACPCs-800. This result is consistent with Xie’s work.^21^ KOH serves as the activating agent for developing mesopores and micropores in the walls of macropores to form an interconnected pore network structure. Song ^26^ performed a detail study on different concentrations of KOH. Therefore, one concentration of KOH was chosen according to Song’s work, and it is enough to develop mesopores.


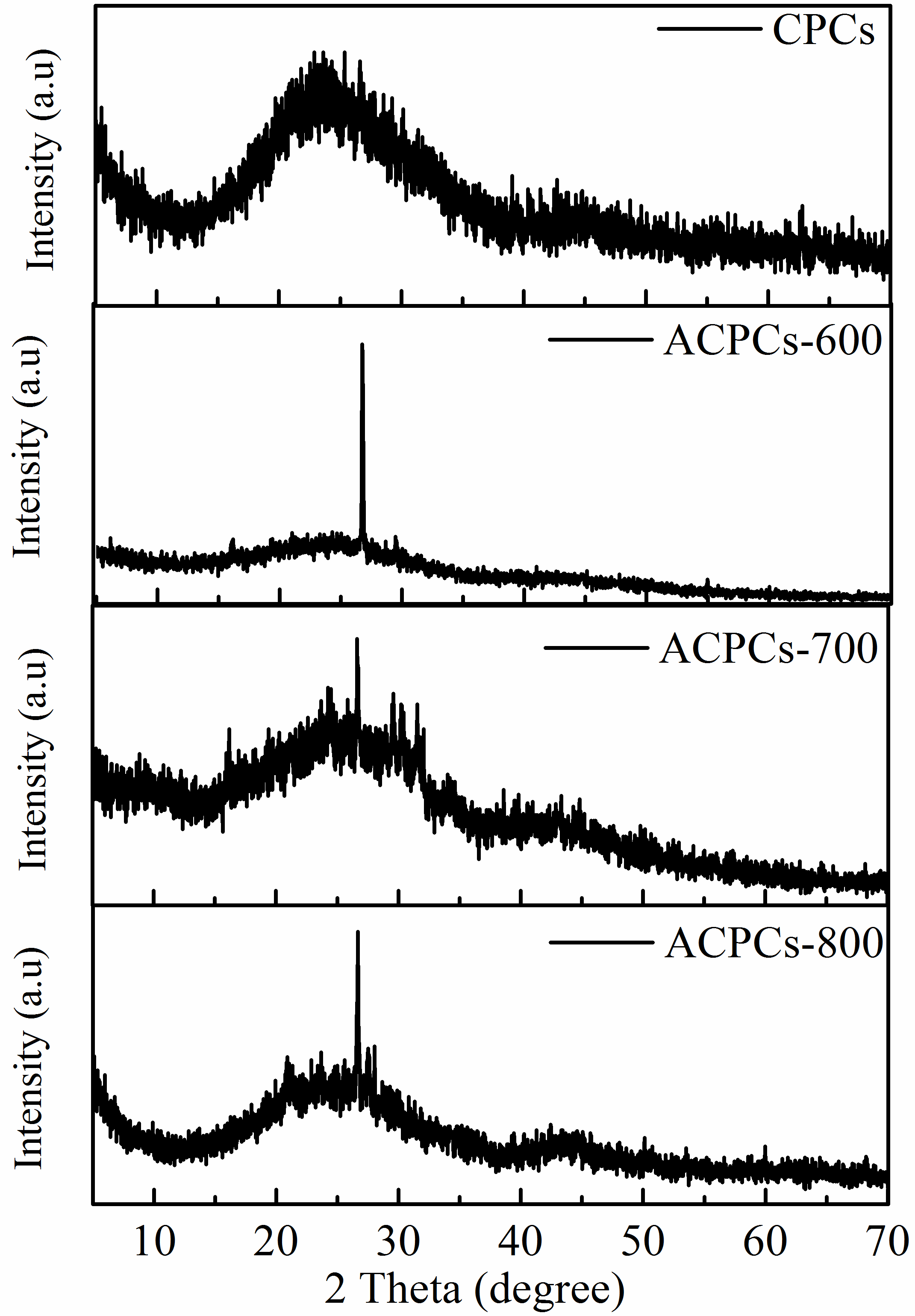


Figure S4 XRD spectra for CPCs and ACPCs-600, ACPCs-700 and ACPCs-800.

Table S1 Concentrations of the concerned elements

| Samples | Ultimate analysis (wt%) | | | | |
| --- | --- | --- | --- | --- | --- |
|  | C | H | N | O | ash content |
| ACPCs | 57.76 | 2.38 | 0.46 | 26.61 | 17.41 |
| CPCs | 64.92 | 2.43 | 1.59 | 21.46 | 8.6 |

Table S2 Characteristics of biochars produced from different feedstocks.

| Feedstocks | Pyrolysis  temperature  (^0^C) | BET surface area (m^2^/g) | Pore volume (cm^3^ /g) | References |
| --- | --- | --- | --- | --- |
| bamboo charcoal | 700 | 67.80 | 0.024 | [1] |
| Pinewood | 700 | 29 | 0.13 | [2] |
| Oak bark | 450 | 1.9 | 1.06 | [3] |
| Tire rubber | 800 | 50 | 0.11 | [4] |
| Rapeseed  plant | 800 | 19 | 1.15 | [5] |
| Spruce  wood | 525 | 40.4 | _ | [6] |
| CPCs | 800 | 191 | 0.10 | This work |
| ACPCs | 800 | 351 | 0.17 | This work |

TableS3 Parameters for Kinetics Models for the Adsorption of Naphthalene and CAP

| Adsorbate | pseudo-first-order | | pseudo-second-order | | | | Intra-diffusion model |
| --- | --- | --- | --- | --- | --- | --- | --- |
| Naphthalene | k_1_(_g mg_^−1^ _min_^−1^) | 0.05 | k_2_ | 8.7*10^-5 | First step | k_3_ 48 b_3_ 87 R^2^ 0.98 | |
|  | *q_e_* (mg g^-1^) | 435 | *q_e_* | 555 | Second step | k_4_ 10.8 b_4_ 322 R^2^ 0.97 | |
|  | R^2^ | 0.83 | R^2^ | 0.99 |  |  | |
| CAP | k_1_(_g mg_^−1^ _min_^−1^) | 0.05 | k_2_ | 5.6*10^-4 | First step | k_3_ 17.2 b_3_ 38.9 R^2^ 0.93 | |
|  | *q_e(mg g_*^-1^*)* | 134 | *q_e_* | 151 | Second step | k_4_ 2.9 b_4_149.7 R^2^ 0.97 | |
|  | R^2^ | 0.91 | R^2^ | 0.99 |  |  | |

Table S4 Structure and properties of organic pollutant

| Pollutants | Chemical  formula | Molecular  weight  (g/mol) | Molecular  Size (nm) | Molecular  Structure | Nature | | water  solubility  (mg/L) |
| --- | --- | --- | --- | --- | --- | --- | --- |
| MB | C_16_H_18_ClN₃S | 319.86 | 0.63 |  | Positive | | 100000 |
| MO | C_14_H_14_N_3_SO_3_Na | 327.33 | 0.129 |  | Negative | | 14850 |
| CR | C_32_H_22_N_6_Na_2_O_6_S_2_ | 696.68 | - |  | Neutral | | 50000  (80^0^C) |
| CAP | C_11_H_12_C_l2_N_2_O_5_ | 323.132 | 4.36 |  | Neutral | 2500 | |
| Naphthalene | C_10_H_8_ | 128.18 | 0.62 |  | Neutral | 30 | |

[1] P. Liao, Z. Zhan, J. Dai, X. Wu, W. Zhang, K. Wang, S. Yuan, Adsorption of tetracycline and chloramphenicol in aqueous solutions by bamboo charcoal: A batch and fixed-bed column study, Chemical Engineering Journal, 228 (2013) 496-505.

[2] Liu, Z., Zhang, F.S., Wu, J., 2010. Characterization and application of chars produced from pinewood pyrolysis and hydrothermal treatment. Fuel 89, 510–514.

[3] Mohan, D., Rajput, S., Singh, V.K., Steele, P.H., Pittman Jr., C.U., 2011. Modeling and evaluation of chromium remediation from water using low cost bio-char, a green adsorbent. J. Hazard. Mater. 188, 319–333.

[4] Lian, F., Huang, F., Chen, W., Xing, B., Zhu, L., 2011. Sorption of apolar and polar organic contaminants by waste tire rubber and its chars in single- and bi-solute systems. Environ. Pollut. 159, 850–857.

[5] Karami, M., Clemente, R., Jimenez, E.M., Lepp, N.W., Beesley, L., 2011. Efficiency of green waste compost and biochar soil amendments for reducing lead and copper mobility and uptake to ryegrass. J. Hazard. Mater. 191, 41–48.

[6] Kloss, S., Zehetner, F., Dellantonio, A., Hamid, R., Ottner, F., Liedtke, V., Schwanninger, M., Gerzabek, M.H., Soja, G., 2012. Characterization of slow pyrolysis biochars: effects of feedstocks and pyrolysis temperature on biochar properties. J. Environ. Qual. 41, 990–1000.
